# Solutions for reducing lawsuits in orthopedic surgery by using psychology and IT technology

**Published:** 2015

**Authors:** VL Purcarea, C Cazac

**Affiliations:** * “Carol Davila” University of Medicine and Pharmacy, Bucharest, Romania

**Keywords:** orthopedic surgery, anxiety, lawsuit, post-traumatic stress disorder, IT technology

## Abstract

Orthopedic surgery is among the top 5 medical specialties with an increased risk of facing a lawsuit. A large part of medical malpractice claims are due to poor communication between physician and patient; therefore, by addressing this issue and implementing psychological methods as well as IT solutions, a reduction in the incidence of medical lawsuits can be achieved. Some of these solutions include implementing and applying psychometric tools such as the SF-36 and SCL-90R tests, creating virtual information hubs for the patient, and establishing efficient communication methods by using IT technology between physician and patient.

## Introduction

In 2011, Anupam et al. published a study in the New England Journal of Medicine that has placed the specialty of orthopedic surgery among the top 5 medical specialties with an increased risk of facing a lawsuit [**1**]. The same study estimated that 99% of physicians practicing specialties with an increased risk of facing a lawsuit will be accused of malpractice at least once before reaching age 65 [**1**]. Furthermore, it is estimated that over 15% of all orthopedic surgeons are annually taken to court [**1**]. 

Although current epidemiologic studies suggest that the incidence of mental disorders of any nature in the general population is of almost 20% [**2**,**3**], it is estimated that in the case of patients who have suffered trauma and were subsequently subject to a surgical intervention, the incidence of mental disorders exceeds the value of 20% [**3**]. For example, Bhandari et al. have discovered a 22% incidence of mental disorders among 215 patients admitted to the hospital with trauma and who were in need of orthopedic surgery. Mason et al. have studied 210 orthopedic trauma patients and have monitored them for 18 months following surgery finding that 30% of them satisfied the criteria necessary for the positive diagnosis of a mental disorder [**3**,**4**]. Another study that lasted for 24 months conducted on patients with severe lower limb problems has shown an incidence of 42% of mental illness for which only 22% have sought medical attention [**3**,**5**].

The relationship between stress induced mental disorder, post-traumatic stress disorder and their effect on an orthopedic patient have long been studied and many authors concluded that the existence of a mental disorder in an orthopedic patient is often associated with a negative post-operative prognosis [**3**,**6**,**8**-**11**].

This article tries to highlight the psychological factors that have a significant pre- and post-operative contribution to the overall medical development of the orthopedic patient. It also tries to show how disease, orthopedic treatment, and subsequent disabilities associated with treatment can lead to stress induced mental disorder. The third goal of this article is to suggest ways in which IT technologies and psychological tactics can contribute to mediating the negative psychological impact associated with the disease. By reducing the psychological distress of the patient there can be a significant decrease of the patient’s desire to file a lawsuit. 

## Discussion

Many current specialty articles have been concerned with finding the main reasons for which orthopedic surgeons are accused of malpractice [**12**,**13**]. One recurrent theme in all these articles was a lack of efficient communication between physician and patient. Beth Huntington from the Office of Risk Management, Baylor Health Care System, mentioned that communication gaffes are the root cause of malpractice claims [**12**].

Generally, the judiciary commissions that had the role of reviewing cases of medical malpractice have recommended orthopedic surgeons to “engage in a thorough pre-operative discussion with the patient (or the family of the patient in the case of children) about the nature of the illness and the proposed treatment, available alternatives, possible complications, consequences of such complications and realistic expectations” [**14**]. These same commissions have underlined the fact that a complete pre-operative discussion between physician and patient is crucial. The frustration of the patient and the absence of a truly informed consent are the most frequent problems that are remarked in cases of medical malpractice lawsuits [**14**]. 

It is not an exaggeration to state that the majority of lawsuits based on malpractice claims could have been avoided if there was an efficient patient-physician communication. For the existence of good communication, it is very important that an orthopedic surgeon understands the psychological factors that affect the patient both pre- as well as post-surgery. 

Another recurrent theme in these studies was the stress associated with the residual disability imposed by the presence of the disease or of the treatment itself. This disability is more evident in the case of illnesses affecting the lower limbs, such as lower limb fractures, which have the potential to make routine daily activities hard or impossible to perform. An article published in collaboration with the American Academy of Orthopedic Surgeons showed that patients who have suffered femur fractures frequently file lawsuits [**14**]. The majority of lawsuits filed by this group of patients were filed on the basis of improper fixation, premature extraction of orthopedic devices and malrotation [**14**]. 

**Preoperative psychological factors affecting patients**

Calvin et al. have conducted a study of 106 orthopedic surgery patients who spanned 3 different generations (young, adults, and elderly) and showed that all 3 age groups presented with moderate to high levels of pre-operative anxiety that started approximately two weeks before surgery [**15**]. Similarly, Gemma Robleta et al. have concluded that preoperative anxiety is the most frequent emotional factor prevalent in patients (72%) and that it represents a predictive factor for the occurrence of moderate to severe post-operative pain [**16**]. 

**Fig. 1:**
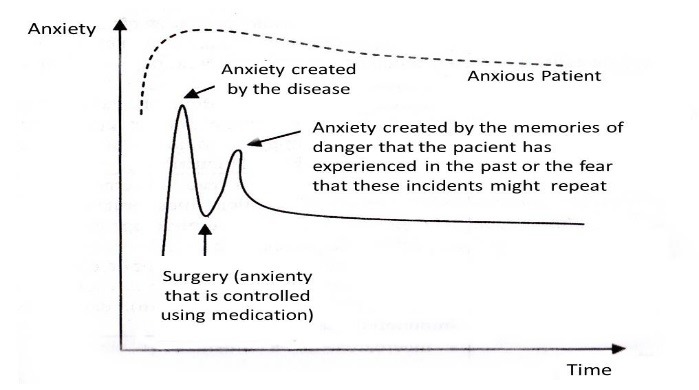
The anxiety profile of patients undergoing emergency surgery. Reproduced and translated from Stiintele Comportamentului Uman: Aplicatii in Medicina, Boli Chirurgicale, Ovidiu Popa Velea [**18**]

**Fig. 2:**
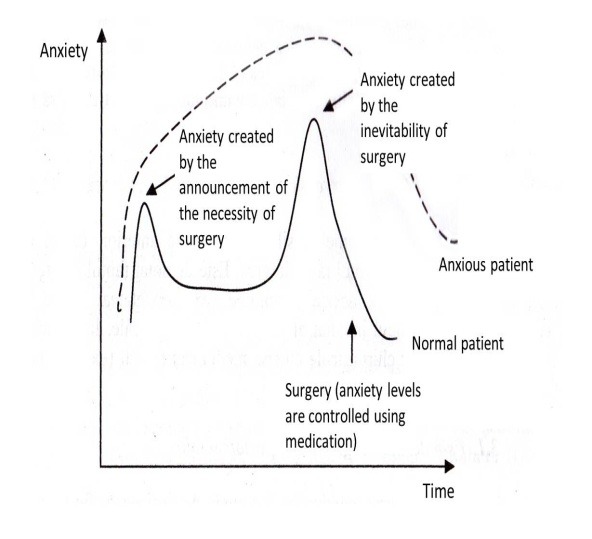
The anxiety profile of patients undergoing pre-scheduled surgical intervention. Reproduced and translated from Stiintele Comportamentului Uman: Aplicatii in Medicina, Boli Chirurgicale, Ovidiu Popa Velea [**18**].

Elaborating on this subject, Ovidiu Popa Velea described the anxiety levels due to surgery found in patients undergoing emergency surgery (**Fig. 1**) and compared them to those found in cases of announced surgical intervention (**Fig. 2**).

Given the differences in anxiety levels between the two profiles above, it becomes clear that each type of patient is in need of a personalized approach toward the management of his or her levels of anxiety. It is important that the orthopedic surgeon understands the anxiety levels and profiles of each patient in order to implement the appropriate medical approach to reduce its levels. 

Generally, the level of pre-operative anxiety needs to be reduced in order to increase the successful rate of recovery after surgery. Laward L and Azar N have shown that a solution to efficiently reduce anxiety levels is represented by the unobstructed access of the patient to information pertaining to his disease, the causes of its appearance as well as to available treatment options written in a way that is understandable to the patient [**18**]. 

To facilitate the access of the patient to information, the majority of hospitals in the U.S.A. and in Europe started to provide basic health information to patients with the help of IT technologies. For example, in the Orthopedics and Joint Replacement department of Mercy Medical Center in Baltimore, Maryland, each physician has a dedicated section on the general website of the hospital www.mdmercy.com. This medical portal contains an abundance of medical information about procedures that each physician can perform. On the webpage of the same orthopedic department, there is a multitude of medical articles, which explain numerous pathologies such as avascular necrosis, bursitis, tendinitis, osteoarthritis, patelo-femural syndrome etc., written in an easy to understand language for patients [**19**,**20**]. To further reduce anxiety levels to a minimum during medical consults, Mercy Medical Center has implemented IT solutions in every orthopedic examination room as well, such as computers connected to a single LAN that can easily show 3D models of treatment to the patient and show the results of medical investigations such as current or previous X-rays, CTs and MRIs. Every examination room contains medical brochures with information about the physician, the diseases he or she treats, as well as advantages and disadvantages of one procedure versus another. All these measures have the direct effect of reducing pre-operative stress and anxiety long before the time of surgery. 

**Negative Post-Operative Psychological Manifestations**

Among the main negative psychological effects that can appear post-surgery are post-traumatic stress disorder (PTSD), post-operative delirium and dependence of opioids [**21**]. Zatzick et al. discovered an incidence of 30% of PTSD in 101 patients admitted to the emergency and trauma departments respectively. Starr et al. conducted an extensive study of 588 patients and found a 51% incidence of PTSD in trauma patients [**7**,**3**]. If we consider PTSD a prototypic example of psychological disorder common in orthopedic trauma patients, then there is a noticeable gap of 31% between the incidence of psychological disorders in the general population (20%) and the high incidence of PTSD in trauma patients (51%). 

Several methods of intervention to reduce the psychological impact of the disease and its treatment have been developed [**17**]: 

1) Recognizing the mental disorder or the significant negative psychological impact of the disease and/ or treatment by the patient and physician 

2) Accepting the emotional reactions of the patient with empathy and avoiding cynical, brutal or indifferent attitudes

3) Trying to offer competent and empathic solutions to the problems of the patient 

4) Recognizing and avoiding sources of conflict 

5) Recognizing medical and personal limits and referring patients to psychological counseling when needed 

**Useful psychometric instruments in the detection of psychological problems of orthopedic patients**

In order to monitor the psychological state of the patient both before and after surgery, psychometric instruments like Symptoms Checklist 90 Revised (SCL-90R) or Short Form 36 (SF-36) can be used [**3**]. 

The SCL-90R is a test that contains 90 questions and is used to monitor the psychological symptoms of patients [**3**,**22**]. This test is useful because it evaluates psychological symptoms on 9 different levels: somatization, obsessive-compulsiveness, interpersonal sensibility, phobic anxiety, paranoid ideas and psychoticism [**3**]. The average time length for completing the test is 15 minutes. 

The SF-36 is a shorter test than the SCL-90R comprising only 36 questions spread across 8 levels that are evaluated separately [**23**]. These 8 levels are: vitality, physical functioning, perception of physical pain, general perception of health, fulfillment of physical role, fulfillment of social role and general mental health [**23**]. Each section is graded differently with a score ranging from 0 to 100 [**23**]. A high score reflects a high functional incapacity. 

Among the major advantages of the SCL-90R and SF-36 is the fact that these tests can be administered fast either when the patient is present at the hospital or when the patient is at home by using a computer. For example, at the moment of the writing this article, the SF-36 test could be found on the official SF-36 website www.sf-36.org/demos/SF-36.html. The results of the test can be easily sent to the orthopedic surgery department to regularly monitor the psychological state of the patient. 

The score obtained on these tests can serve to monitor the psychological state of the patient both in the pre-operative stage as well as in the post-operative stages of recovery. By using these powerful tools, patients with a high risk of psychological distress can be detected and the appropriate action, such as reducing anxiety levels and stress, can be implemented. 

**Results of SCL-90R and SF-36 in high-risk patients**

The results of Bondhari’s study have shown that patients who exhibited distress and who were administered the SCL-90R test were usually showing increased levels of phobic anxiety and somatization [**3**]. In addition, the same study showed that one in 5 patients was meeting the score criteria for mental illness [**3**]. Patients who were administered the SF-36 checklist and who presented with high scores exhibited the following characteristics: high intensity of psychological symptoms, the preferential localization of the fracture on the lower limbs, ongoing litigation and older age [**3**]. 

These results showed the fact that patients with an increased risk of psychological distress are the patients who present the following characteristics: localization of the disease in the lower limbs which is associated with a greater degree of disability, ongoing litigation, older age, the presence of somatization and phobic anxiety. As a result, patients presenting these risk factors need to be strictly monitored both in the preoperative setting as well as during post-operative recovery to ensure psychological comfort. This increased level of care will eventually lead to a better patient-physician relationship and dramatically reduce the chances of facing a lawsuit. 

**The role of IT technology in detecting, monitoring and eliminating psychological risk factors**

The SF-36 and SCL-90R tests can be administered both when the patient is in a hospital setting as well as when the patient is at home during recovery. These tests can be performed online and the results can be sent directly to the physician. The physician can then decide if the patient is in good psychological health, in need of increasing psychological comfort or in need of a referral to a psychologist. 

Another way in which IT technologies can contribute to reducing pre-operative anxiety is represented by the availability of health care information online written in a language that can be easily understood by the patient. Therefore, the web page of the hospital must be primarily oriented toward the easy access of the patient. This web portal needs to include the professional qualifications of the physician to increase the patient’s trust in the physician’s abilities while simultaneously reducing pre-operative anxiety. Also, the web page of the hospital needs to include information about the illnesses that are regularly treated in the specialized departments of the hospital. 

IT technologies can also significantly contribute to increasing the patient-physician relationship by using a dedicated email platform that can be used by both the patient and the physician. By the use of this dedicated platform, patients can ask or report to the physician about any developments without interrupting the physician from his activities. The physician can answer patient emails when he is available for this activity thus maintaining a good communication even when the patient is not in the hospital. As Theodore J. Clarke, MD, mentioned, orthopedic surgeons who have never been sued excel in communication techniques with their patients [**24**]. 

## Conclusion

Orthopedic surgery is among the top 5 medical specialties with an increased risk of facing a lawsuit. A large part of medical malpractice claims are due to poor communication between physician and patient; therefore, by addressing this issue and implementing psychological methods as well as IT solutions, a reduction in the incidence of medical lawsuits can be achieved. Some of these solutions include implementing and applying psychometric tools such as the SF-36 and SCL-90R tests, creating virtual information hubs for the patient, and establishing efficient communication methods by using IT technology between physician and patient. 

Purcarea VL and others showed that because of the rapid expansion of IT technologies, patients have developed a tendency to appreciate the quality of healthcare based on perceived expectations and not based on reality [**25**-**27**]. This is the primary reason why it would be useful to direct efforts toward the optimization of IT technologies in the hospital setting, increasing communication between patients and medical personnel, exploring patient behavior, addressing and intervening when a patient is presenting with psychological distress, remodeling the hospital infrastructure so that it offers an integrated flow of information, and finally of investigating the crucial impact that virtual communities have on the reputation of the healthcare provider [**26**-**27**]. 
